# Spatial Variability of Soil Properties and Portable X-Ray Fluorescence-quantified Elements of typical Golf Courses Soils

**DOI:** 10.1038/s41598-020-57430-y

**Published:** 2020-01-16

**Authors:** Yujian Yang, Xueqin Tong, Yingpeng Zhang

**Affiliations:** 1 S&T Information Institute of Shandong Academy of Agricultural Sciences, Agronomy College of Shandong University, Jinan, China; 2 S&T Information Institute of Shandong Academy of Agricultural Sciences, Jinan, China; 3 Institute of Agricultural Resources and Environment of Shandong Academy of Agricultural Sciences, Jinan, China

**Keywords:** Ecology, Environmental sciences

## Abstract

Understanding and quantitative delineation of Portable X-Ray Fluorescence (PXRF) -quantified elements and soil properties spatial variability are important for healthy turf development for golf courses. In this study, PXRF-quantified elements and soil properties (except soil acidity and alkalinity (pH), electric conductivity (EC), and textures) of 200 soil samples were measured by PXRF analyzer at different golf courses in Lubbock, Amarillo, and Midland in Texas, and Hobbs in New Mexico. Furthermore, principal component analysis (PCA), empirical bayesian kriging (EBK) and the ordinary least square model (OLSM) were used in the study. Two kinds of components were extracted and interpreted by PCA, the results showed Zn, Ti, Fe, Rb, V, Mn and Zr were associated with the component 1, while Sr was associated with the component 2, the preliminary classification of PXRF-quantified elements was formed by PCA. The EBK approach was used to evaluate the spatial patterns of PXRF-quantified elements and soil properties. The OLSM model quantitatively related pH to EC, silt texture and the PXRF-quantified K, Ca and Sr. The integration of PCA, EBK and OLSM revealed quantitative links between soil pedogenesis and causes, spatial variability and couple relationships of PXRF-quantified elements and soil properties over golf courses.

## Introduction

Currently, proximal soil sensing presents the characteristics of interdisciplinary with pedometrics, pedology and morphology. Proximal and remote sensing, along with miniaturized sensor technology, have advanced the useful non-destructive monitoring technologies for rapidly quantifying heavy metal concentrations. The Portable X-ray Fluorescence (PXRF) spectrometry is a proximal scanning technology and has a long history of being used in characterizing the elemental compositions in many matrices^1,2^. The PXRF technology can be implemented in soil geochemical analysis for fast and efficient testing of heavy metals^3^, and used to answer the questions of direct soil provenance based on the understanding that both soil pedogenesis with climate, parent material, topography and other factors^4^. Applications of PXRF included environmental assessment and identification of heavy metal concentrations of soils, compost, or solid waste^5–7^. PXRF applications to pedology were rapidly increasing given the relative ease of data acquisition^8^. The PXRF’s ability to predict soil textural attributes was demonstrated, which is of interest in texture related to mineral composition^7^. PXRF was valid as well to rapidly screen Cr and Ni levels in serpentine soils^3^. Laiho and Perämäki (2005) found that soil moisture and particle size were the main factors influencing on PXRF measurement accuracy from contaminated site soils^9^. The response of X-ray fluorescence (XRF) intensity to soil moisture content depended on soil texture and mineralogy^10^, however, it is generally stable in measuring soil elements when moisture content is <15%^8,11^.

Over the past 10 years, PXRF has been much more used in soil science. Among many contemporary methods which sanction the use of PXRF for soil analysis, the US Environmental Protection Agency’s (EPA) Method 6200:“Determination of Elemental Concentrations in Soil and Sediment”^12^ is one of the most widely cited. Additionally, using PXRF has also been found in the Soil Survey Field and Laboratory Methods Manual by the US Department of Agriculture, Natural Resources Conservation Service^13^. The advantages of PXRF for rapid assessment of soil heavy metals were demonstrated by Kalnicky and Singhvi^14^. 17 soil samples using PXRF from abandoned mining sites were evaluated by Radu and Diamond^2^ in Ireland. An on-the-go spectrometer for *in situ* measurement and prediction of various soil properties were developed by Christy^15^. The PXRF suitability to extract paleo-geochemical information from lacquer-peel soil sections that have been taken to document pedological information at archaeological sites was demonstrated by Arnoldussen and Vanos^16^. Lithogenic and pedological evolution processes were reflected by the values for Si, K, Al, Fe, Ti, Sr, Zr, and Rb in the lacquer peels, while the contents of Ca, S, and P were used as a proxy for anthropogenic influence. Similarly, Weindorf *et al*. (2012) examined lithogenic discontinuities in soils using PXRF^5^. Many previous applications of PXRF have focused on heavy metal concentrations for screening, monitoring, mitigation, and environmental assessment^2,17–21^. Few studies have performed spatial variability of soil properties and PXRF-quantified elements in semi-arid soils for golf courses. In many instances, PXRF applications replaced traditional laboratory analysis showing great promise given its portability, low cost, fast speed, and minimal requirements for sample preparation^8,22,23^. It is apparently useful to couple PXRF with spatial analysis techniques to assess spatial variability of PXRF-quantified elements and soil properties.

Evidently, spatial analysis has been progressing to increase prediction accuracy of soil properties and PXRF-quantified elements. Recently, model-based geostatistics, Sequential Gaussian Simulation (SGS) and Sequential Indicator Simulation (SIS) have also been used to predict and map soil contamination, and alternative approaches are to use the bayesian maximum entropy (BME)^24^ (D.J.Bus *et al*., 2008), spatial copula method, and robust geostatistics. However, empirical bayesian kriging (EBK) works fine, and gives good results. It is a geostatistical interpolation approach, and differs from other kriging methods used by many semivariaogram models which examine the spatial autocorrelation between the measured sample points from the different directions and distances rather than a single semivariogram^25^. Then, semivariogram weight is calculated using the Bayes’ rule, which shows the likelihood of measured data generated^25,26^. EBK automates the model parameters through simulation and, and generates simulation results based on data non-stationary characteristics. Though EBK calculation is slower than other kriging methods, it is more accurate for small datasets (for instance, the EBK maps of PXRF-quantified elements and soil properties in the study) in practice^27^. Samsonova *et al*. (2017) developed a comparison study of organic carbon content using ordinary kriging and EBK, showed that EBK had good performance by revealing the heterogeneities of soil properties and PXRF-quantified elements^28^. Therefore, we chose EBK approach to generate prediction maps of soil properties and PXRF-quantified elements in golf course soils.

Golf is associated with several benefits, e.g. it provides recreational value for the many people who play the game, enhances local biodiversity through extensively managed roughs in areas. A set of study about soil pedogenesis and causes, spatial variability and couple relationships of the PXRF-quantified elements and soil properties over golf courses is more beneficial to improve intensively turfgrass management. As such, the specific objectives and aims of this research were to: (1) sample and scan soils from golf courses, and develop spatial variability of them via EBK; (2) Moreover, perform coupled relationships between soil properties and PXRF-quantified elements based on OLSM, and reveal the quantitative relationship between lab measured variables and PXRF-quantified elements from the soils sampled in golf courses.

## Results

### Data statistical and OLSM analysis

Descriptive statistics of the 200 soil samples are presented in Table 1. Soil pH values ranged from slightly acid (6.97) to moderately alkaline (8.35) with a slightly alkaline mean (7.72). Soil EC and PXRF-quantified elements (Ca, Zn, V, Mn, Fe and Sr) featured larger ranges. Generally, there are three classes concerning coefficient of variation (CV): weak variation (CV < 0.1), medium variation (0.1 <  = CV <  = 1.0), and strong variation (CV > 1.0). According to variation classification, the results revealed weak to moderate variability in pH, EC and PXRF-quantified elements. The CV of pH was weak (0.050), EC was medium (0.783), and PXRF-quantified elements displayed medium variation with Ca (0.936), K (0.404), Zn (0.778), Ti (0.457), Fe (0.549), Rb (0.444), V (0.523), Mn (0.608), Zr (0.398) and Sr (0.662). The aforementioned elemental variation cannot illustrate the spatial structural characteristics and the random variation of soil properties and PXRF-quantified elements. Consequently, it is necessary to apply geostatistical methods to uncover a detailed structural and random characteristics.

**Table 1:** Descriptive statistics of soil properties and PXRF-quantified elements in Amarillo, Hobbs, Lubbock and Midland (N = 200).

Variable	Range	Min.	Max.	Mean	Std. Deviation	Variance	Skewness	Kurtosis	CV
pH	1.38	6.97	8.35	7.72	0.39	0.15	−0.39	−1.15	0.050
EC	3692.00	198.00	3890.00	1313.18	1027.96	1056700.39	1.07	−0.48	0.783
Zn	314.00	7.00	321.00	58.09	45.17	2040.29	1.66	5.57	0.778
K	1.59	0.37	1.96	1.12	0.45	0.20	0.02	−1.38	0.404
Ca	14.75	0.20	14.95	3.10	2.90	8.43	1.41	1.86	0.936
Ti	0.28	0.05	0.33	0.17	0.08	0.01	0.23	−1.16	0.457
V	70.00	8.00	78.00	36.33	18.99	360.46	0.25	−1.21	0.523
Mn	471.00	26.00	497.00	188.40	114.48	13106.49	0.43	−0.63	0.608
Fe	2.34	0.24	2.58	1.05	0.58	0.33	0.45	−0.68	0.549
Rb	81.70	17.30	99.00	50.32	22.32	497.97	0.20	−1.18	0.444
Sr	536.70	22.300	559.00	198.54	131.44	17277.32	0.63	0.02	0.662
Zr	543.00	127.00	670.00	339.95	135.34	18316.96	0.62	−0.68	0.398

N: Sample numbers. Unit of every variable: EC (dS m^−1^), Zn (mg kg^−1^), K (%), Ca (%), Ti (%),V (mg kg^−1^), Mn (mg kg^−1^), Fe (%), Rb (mg kg^−1^), Sr (mg kg^−1^), Zr (mg kg^−1^).

The data for all the 200 samples in Amarillo, Lubbock, Midland and Hobbs were used to evaluate soil properties and PXRF-quantified elements’ interaction on basis of Pearson’s correlation matrix. We studied with 11 interval-level variables to estimate the relationships among all of them. The result was illustrated in Table 2. Specifically, there was a strongly positive correlations between K and Ti (0.96), K and V (0.96), K and Mn (0.93), K and Fe (0.96), K and Rb (0.97), all with R values > 0.90. The strongest positive correlation was between Fe and Rb (0.99).

**Table 2:** Pearson correlation coefficients for soil properties and PXRF-quantified elements in Amarillo, Lubbock, Midland, and Hobbs (N = 200).

	pH	EC	K	Ca	Zn	Ti	V	Mn	Fe	Rb	Sr
EC	−0.62^b^										
K	−0.13	−0.15^a^									
Ca	−0.06	0.17^a^	0.44^b^								
Zn	−0.13	−0.15^a^	0.77^b^	0.31^b^							
Ti	−0.08	−0.18^a^	0.96^b^	0.36^b^	0.79^b^						
V	−0.05	−0.17^a^	0.96^b^	0.45^b^	0.80^b^	0.98^b^					
Mn	−0.22 ^b^	−0.11	0.93^b^	0.21^b^	0.80^b^	0.93^b^	0.91^b^				
Fe	−0.12	−0.17^a^	0.96^b^	0.29^b^	0.78^b^	0.97^b^	0.95^b^	0.96^b^			
Rb	−0.12	−0.14^a^	0.97^b^	0.37^b^	0.80^b^	0.98^b^	0.97^b^	0.95^b^	0.99^b^		
Sr	−0.54^b^	0.74^b^	0.32^b^	0.49^b^	0.26^b^	0.25^b^	0.28^b^	0.33^b^	0.27^b^	0.33^b^	
Zr	0.09	−0.20^b^	0.76^b^	0.53^b^	0.68^b^	0.83^b^	0.83^b^	0.65^b^	0.71^b^	0.77^b^	0.17^a^

^a^Significant at α = 0.05 level; ^b^Significant at α = 0.01. N: Sample numbers. Unit of every variable: EC (dS m^−1^), K (%), Ca (%), Zn (mg kg^−1^), Ti (%), V (mg kg^−1^), Mn (mg kg^−1^), Fe (%), Rb (mg kg^−1^), Sr (mg kg^−1^), Zr (mg kg^−1^).

A descriptive statistical analysis of pH, K, Fe and Sr was performed with the data measured from Amarillo, Hobbs, Lubbock and Midland, respectively. The mean value statistics indicated that PXRF-quantified K content was 1.58 (%) in Amarillo, 1.43 (%) in Lubbock, 0.90 (%) in Midland, and 0.56 (%) in Hobbs. The biggest mean value was from Amarillo which was almost as three times as the smallest mean value from Lubbock. The highest standard deviation (SD) of Fe concentration was 0.46 (%) in Lubbock, indicating that the sampling data points were spread out over a wider range of the values, while the smallest SD of Fe concentration was 0.09 (%) in Hobbs. There was a similar mean of 200.92 (mg kg^−1^) in Amarillo and 206.76 (mg kg^−1^) in Lubbock for Sr concentration, but their ranges varied markedly from 134.00 (mg kg^−1^) to 274.00 (mg kg^−1^) in Amarillo, from 105.00 (mg kg^−1^) to 548.00 (mg kg^−1^) in Lubbock, and from 66.40 (mg kg^−1^) to 559.00 (mg kg^−1^) in Midland (Table 3).

**Table 3:** Comparative study of typical parameters located in Amarillo, Hobbs, Lubbock and Midland.

Items	Parameters	Amarillo	Hobbs	Lubbock	Midland
**pH**	Mean	7.95	8.07	7.53	7.35
	Minimum	7.69	7.71	6.97	7.08
	Maximum	8.31	8.35	8.30	7.70
	Std. Deviation	0.14	0.15	0.45	0.16
**K**	Mean	1.58	0.56	1.43	0.90
	Minimum	1.28	0.37	0.87	0.58
	Maximum	1.96	0.80	1.96	1.32
	Std. Deviation	0.10	0.10	0.31	0.20
**Fe**	Mean	1.59	0.38	1.49	0.75
	Minimum	1.08	0.24	0.75	0.42
	Maximum	2.35	0.62	2.58	1.04
	Std. Deviation	0.27	0.09	0.46	0.15
**Sr**	Mean	200.92	36.89	206.76	349.59
	Minimum	134.00	22.300	105.00	66.40
	Maximum	279.00	71.40	548.00	559.00
	Std. Deviation	32.73	10.13	80.01	112.13

N: Sample numbers. Unit of every variable: K (%), Fe (%), Sr (mg kg^−1^).

**Figure 1:**
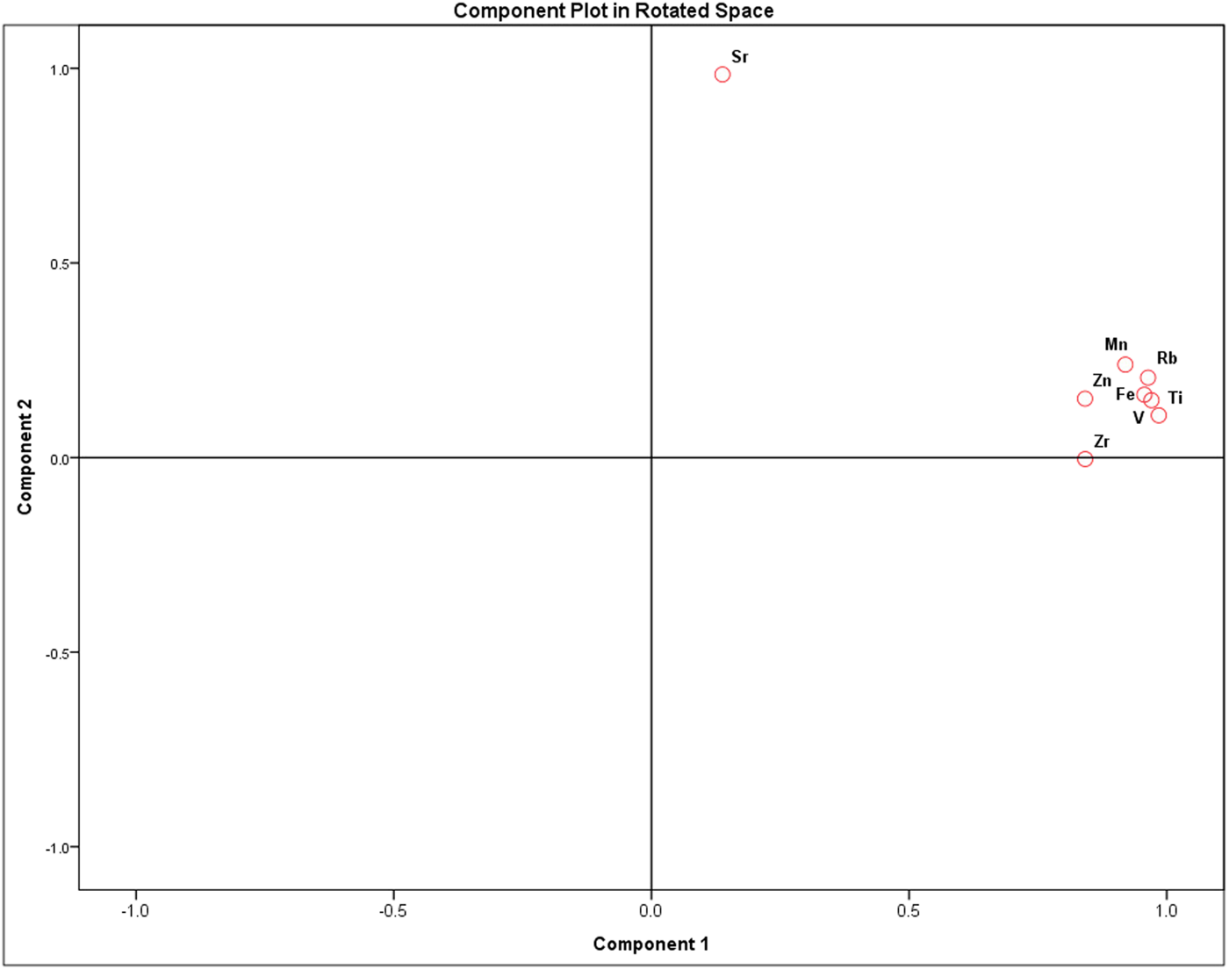
Principal component analysis of PXRF-quantified elements at 4 golf courses in Amarillo, Hobbs, Lubbock and Midland.

Particularly, we further focused on soil textures (sand, silt, clay) and PXRF-quantified elements to improve the turf located in Lubbock. The descriptive statistical results of the 50 collected soil samples in Lubbock showed that sand ranged from 18.5 to 79.2% with a mean of 54.2%, silt ranged from 4.0 to 36.3% with a mean of 16.3%, and clay ranged from 11.8 to 52.4% with a mean of 29.4% and EC, sand, silt, clay, Zn, K, Ca, Ti, V, Mn, Fe, Rb and Sr all presented medium variations with EC (0.299), sand (0.303), silt (0.475), clay (0.396), Zn (0.282), K (0.217), Ca (0.627), Ti (0.205), V (0.244), Mn (0.323), Fe (0.306), Rb (0.250), and Sr (0.387). Generally, PCA generates a new set of variables called principal components, each principal component is a linear combination of the original variables, and all the principal components are orthogonal to each other and no redundant information. PCA loading plots of 8 elements are presented in Fig. 1. Elements grouped into a two-component model accounted for ~89.84% of the total variability, with 75.43% and 14.39% linked to components 1 and 2, respectively. Results showed that Zn, Ti, Fe, Rb, V, Mn and Zr were loaded in principal component 1 and Sr were loaded in principal component 2. The PCA is a method commonly used in geochemical applications to mostly define natural and anthropogenic pollutant sources (T. K. Udeigwe, *et al*.; Eze *et al*.)^29,30^. Evidently, anthropogenic disturbance easily affected spatial variability of soil properties and PXRF-quantified elements, especially at surface 0–10 cm soil. Sr is one of the most abundant elements in the earth crust, Sr concentration was profoundly influenced by anthropogenic activities in golf course soils. Though the two associations of PCA indicated two different possible pollutant sources, whether Zn, Ti, Fe, Rb, V, Mn and Zr could be originated from the similar lithology origin, or Sr element concentration was more likely linked to the contaminants from anthropogenic activities (e.g., traffic, management practices), needs further verification by additional evidence, or measured data.

**Figure 2:**
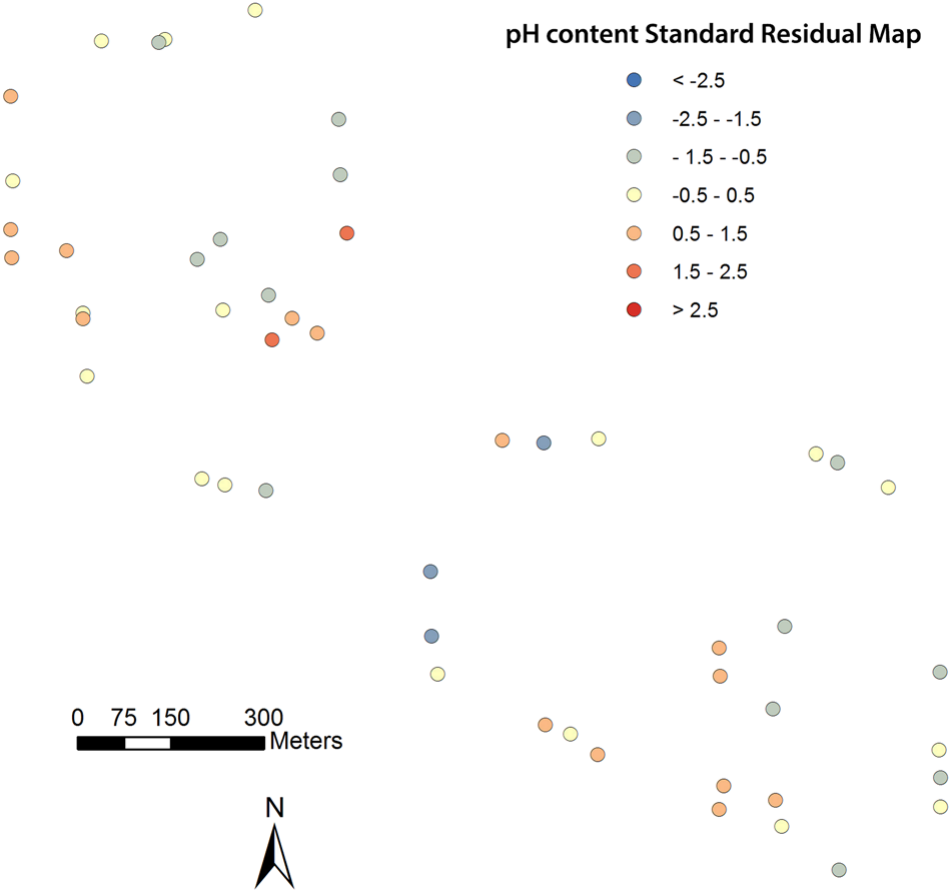
Standard residual map of pH variable based on OLSM in Lubbock.

This preference for OLSM stems from its simplicity and ease of use, computational efficiency, and straightforward interpretation, which involved the dependent and explanatory variables selected as well as the spatial weights to perform the model computerization^31,32^. To clarify the relationship between soil properties and PXRF-quantified elements, we performed OLSM which was constructed between pH (dependent variable) and soil elements (explanatory variables). Concretely speaking, pH was dependent variable, EC, silt texture, PXRF-quantified K, PXRF-quantified Ca and PXRF-quantified Sr were the explanatory variables with their statistical significances. The following relationship was found between the dependent variable and the explanatory variables:

1 $${\rm{pH}}=-\,0.00063{\rm{EC}}-0.01169{\rm{silt}}-0.00022{\rm{K}}-0.00003{\rm{Ca}}+0.00316{\rm{Sr}}+8.4576$$

with adjusted R^2^ = 0.56 (more technically, the model is explaining 56 percent of the variation in the pH content dependent variable), AIC (Akaike Information Criterion) = 30.71 and a significance p < 0.001^32,33^. the lower AIC the measure, the better the fit. A standard residual map may give an indication of systematic over- or under- prediction in particular regions, it clearly illustrated patterns of over- or under-prediction. As illustrated in Fig. 2, the standard residual map of pH variable was generated by the OLSM based on data observed from different soil samples in Lubbock, Texas. Moreover, the mean absolute percentage error (MAPE) was calculated per Eq.(2) to conduct model error statistics:

2 $${\rm{MAPE}}=(\frac{1}{n}\sum \frac{|observed-estimated|}{|observed|})\times 100$$

MAPE of pH was equal to 0.237, which showed estimated pH from OLSM which revealed the quantified-elements and soil properties had better accuracy and practical significance.

### Empirical bayesian kriging

**Figure 3:**
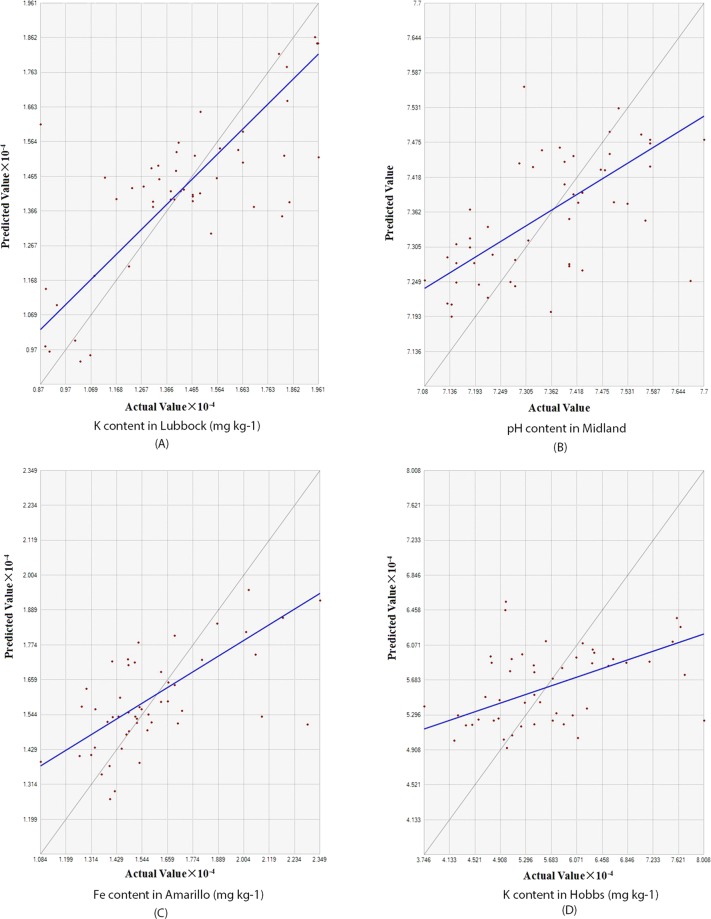
Scatterplots of predicted values and actual values of PXRF-quantified K content in Lubbock (**A**), pH content in Midland (**B**), PXRF-quantified Fe content in Amarillo (**C**), PXRF-quantified K content in Hobbs (**D**).

To verify the prediction, we generated several physicochemical soil properties scatterplots of measured values versus predicted values. These were example scatterplots of the measured values (PXRF-quantified K, PXRF-quantified Fe and measured pH) versus the predicted values across the golf courses. In these scatterplots, the fitted line through the scatter points was given in dark blue, as shown in Fig. 3. (A) to (D), the X-coordinate and Y-coordinate values denoted predicted value and actual values (PXRF-quantified-K content in Lubbock, pH content in Midland, PXRF-quantified-Fe concentration in Amarillo, PXRF-quantified-K content in Hobbs). However, the slope was usually less than 1 because kriging tends to under-predict large values and over-predict small values. The “goodness of fit” accuracy and EBK parameters of quantified-elements and soil properties are given in Table 4.

**Table 4:** EBK parameters of soil properties and PXRF-quantified elements in Amarillo, Hobbs, Lubbock and Midland.

Items	Semiviariogram model	Model Parameters				
		ME	RMSE	ASE	MSE	RMSSE
pH(Lubbock)	Power	0.010	0.373	0.398	0.022	0.956
EC(Lubbock)	Power	−0.073	256.082	263.106	−0.001	0.973
Sand(Lubbock)	Linear	0.046	13.866	14.596	0.005	0.963
Silt(Lubbock)	Linear	0.124	7.989	8.048	0.0156	0.993
Clay(Lubbock)	Linear	0.057	9.593	10.089	0.002	0.963
Zn(Lubbock)	Thin plate spline	−0.072	18.029	19.091	-0.006	0.954
K(Lubbock)	Power	−16.158	2041.92	2213.895	−0.0075	0.951
Ca(Lubbock)	Linear	52.057	7781.003	8083.221	0.001	0.970
Sr(Lubbock)	Power	4.139	76.959	79.736	0.0495	0.969
Fe (Amarillo)	Power	−54.395	2137.838	2261.37	−0.014	0.957
pH (Midland)	Power	0.0025	0.127	0.129	0.013	0.985
K (Hobbs)	Power	−22.424	908.079	914.503	−0.023	0.995

Unit of every variable: EC (dS m^−1^), Sand (%), Silt (%), Clay (%), Zn (mg kg^−1^), K (%), Ca (%), Sr (mg kg^−1^), Fe (%).

**Figure 4:**
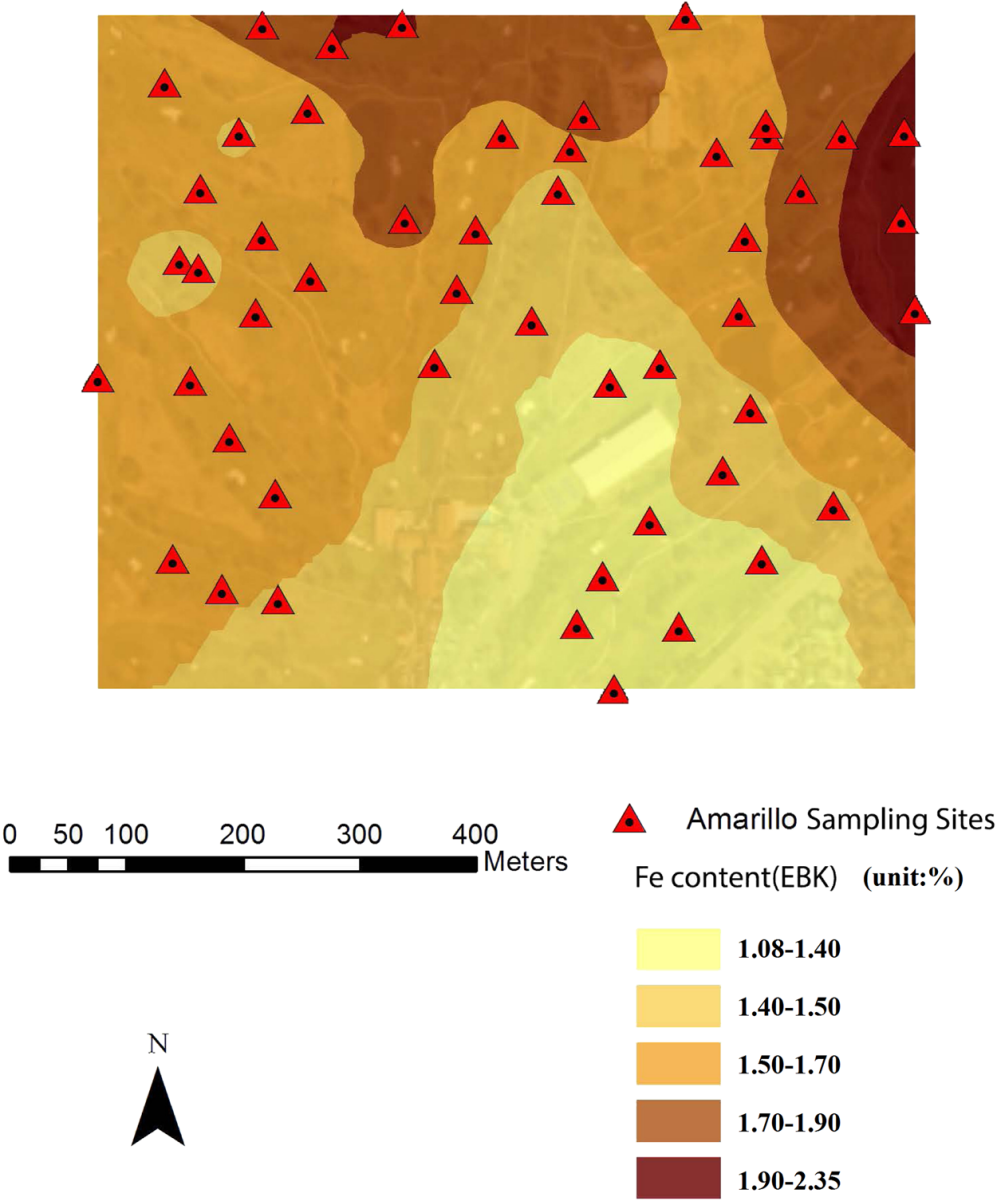
EBK prediction of surface of PXRF-quantified Fe element at a golf course facility in Amarillo, TX, U.S.A.

**Figure 5:**
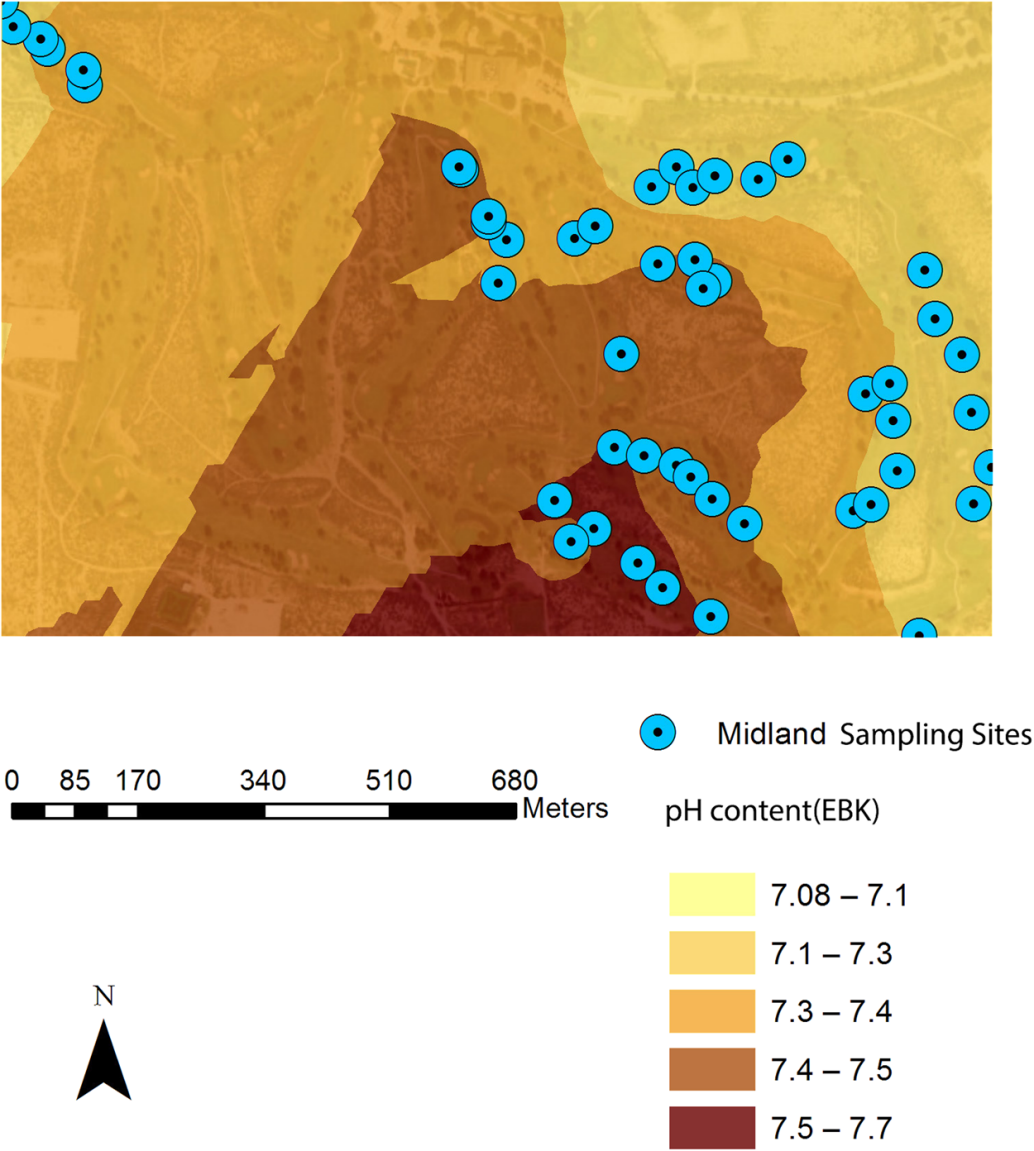
EBK prediction of surface of soil pH at a golf course facility in Midland, TX, U.S.A.

**Figure 6:**
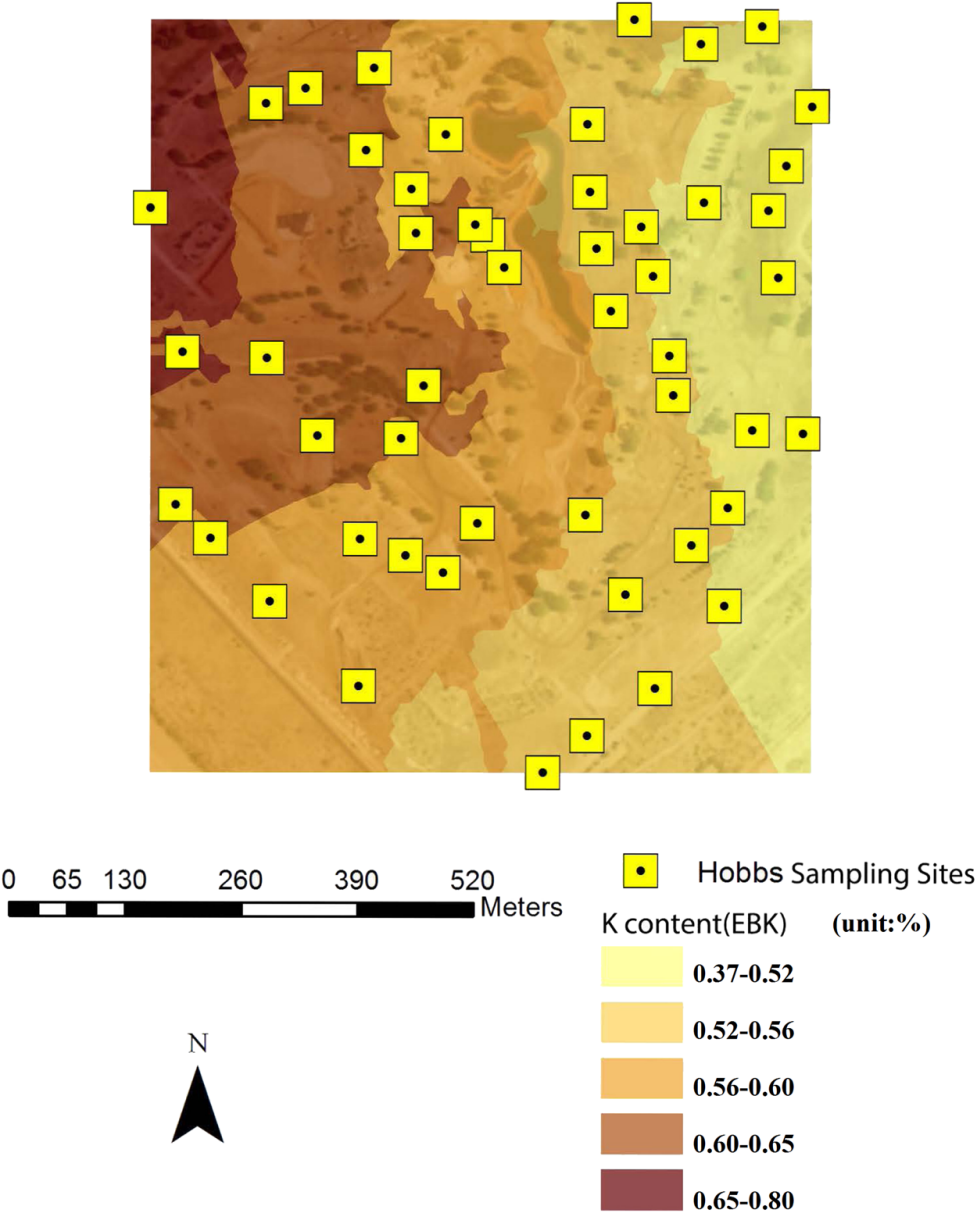
EBK prediction of surface of PXRF-quantified K element at a golf course facility in Hobbs, NM, U.S.A.

**Figure 7:**
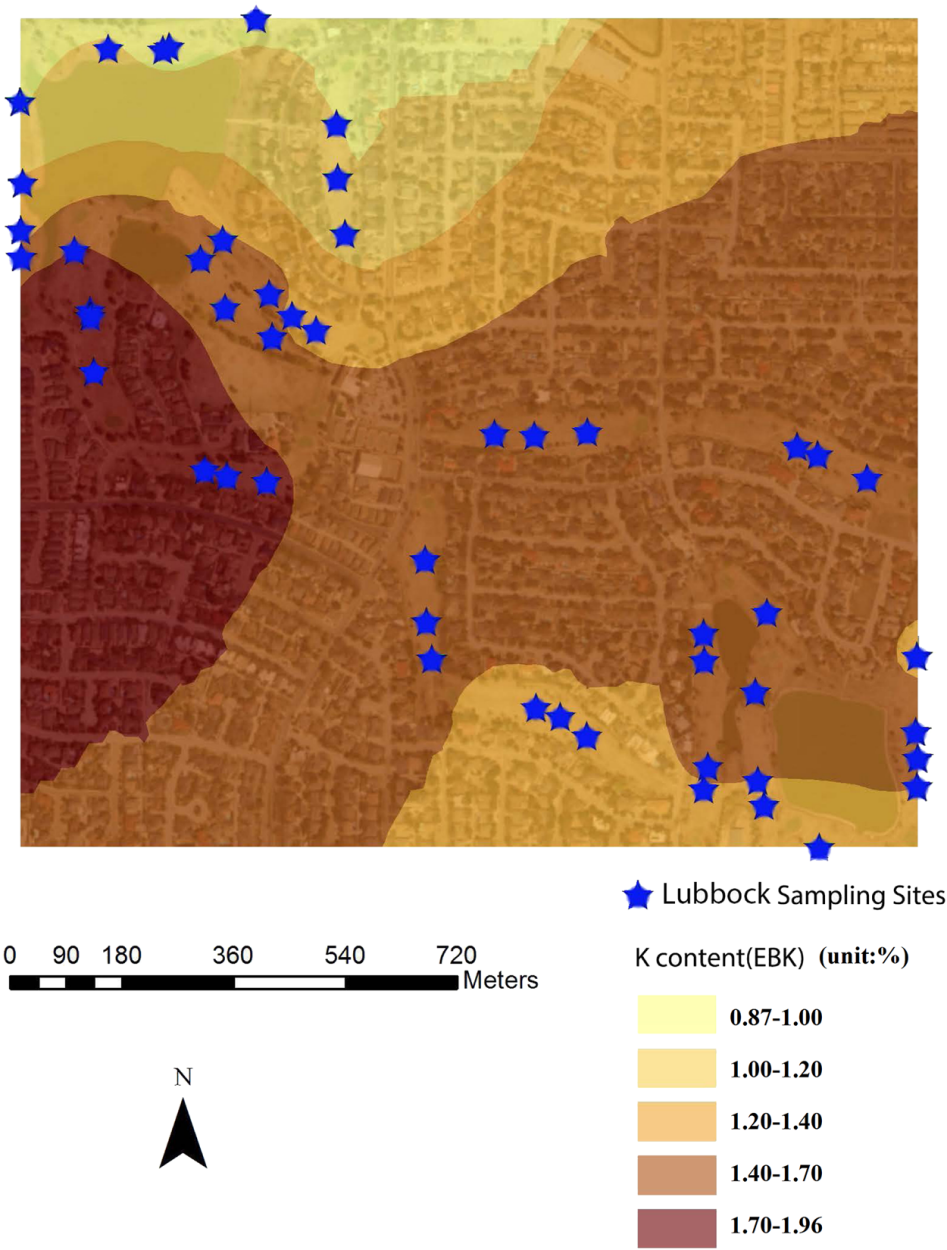
EBK prediction of surface of PXRF-quantified K element at a golf course facility in Lubbock, TX, U.S.A.

Combined with remote sensing image (30 cm resolution), spatial variability maps of soil properties and PXRF-quantified elements were rendered based on EBK with 30% transparency. Figures 4–7 were examples of the variation of physicochemical soil properties and PXRF-quantified elements, were produced by EBK approach. These maps explicitly revealed the spatial patterns which provided more details of the studied elements, and illustrated the low content and high content areas across the golf courses located in Amarillo, Lubbock, and Midland in Texas and Hobbs in New Mexico. From the Fig. 4, PXRF-quantified-Fe concentration was presented from the southeast to the north-west of the golf course. Specifically, the highest concentration of PXRF-quantified Fe was created in the northeast of the Amarillo golf course, and the lowest concentration was created in the southeast of the golf course. Basically, the spatial pattern of the semi-concentric zone was presented from the low concentration to the high concentration of PXRF-quantified Fe, and the variation coefficient of PXRF-quantified Fe concentrations is 0.306 with medium variation from statistical results. The characteristics of PXRF-quantified Fe concentrations was in detail delineated by statistics combined with a geostatistical EBK approach.

### Discussion

A wide range of natural and anthropogenic types and spatial variation of soil contaminants making potentially very high costs^31^, while PXRF spectroscopy is a better method for soil heavy metal remediation providing real-time measurements of quantified-elements (e.g., Zn, Ti, Fe, Rb, V, Mn, Zr and Sr) to support an on-the-go assessment. PXRF applications have the potential to decrease costs. Nevertheless, the detection limit of the PXRF-measured soil elements is an important topic. Quality assurance of PXRF in the study scan data was accomplished via scanning two (NIST: National Institute of Standards and Technology, USA) NIST-certified reference soils (2710a and 2711a)., the NIST values are followed by PXRF-determined values, with all values in mg kg^−1^: 2710a (Mn, 2,140, 2,182; Fe, 43,200, 45,450;Cu, 3,420, 3,258; Zn, 4,180, 4,114;As, 1,540,1,468; Ca, 9,640, 7,850; Pb, 5,520, 5,371; K, 21,700, 24,750; Ti,3,110, 3,514; Sb, 53, 57; Sr, 255, 262) and 2711a (Ti, 3,170, 2,904; As, 107, 73; Ca, 24,200, 23,550; Cu, 140, 112; Fe, 28,200, 21,950; Pb, 1,400, 1,302; Mn, 675, 572; K, 25,300, 23,650; Zn, 414, 342; Sb, 24, 37; Sr, 242, 222) (Samantha Swanhart, *et al*.)^23^. The majority of the elements, reasonably good correlation was found between PXRF and ICP data (As, Co, Cu, Fe, Mn, Pb and Zn), but concluded that ICP analysis provided better detection of elements at low levels (<5 mg kg^−1^) which was related to the detection limit of the PXRF device (Weindorf *et al*.)^5,34^. In practice, some regulatory limits of the metals may not fall above the detection limit of the PXRF device. However, numerous factors have an effect on the detection limits and the precision of the measurements, soil moisture and particle size are the main factors influencing the accuracy of the results (Laiho and Perämäki)^9^. ICP-MS and ICP-OES were used to determine patterns of soil elemental composition for their high level of accuracy (Horta, A. *et al*.)^31^. The comparative study of future work between ICP-MS and PXRF-quantified elements is interesting for golf courses, which can also validate and calibrate heavy metals spectrum accuracy each other for different approaches.

Golf course soils were disturbed by anthropogenic activities including mowing, irrigation and fertilization, aeration, topdressing, particularly on fairways (Pernilla Tidåkera, *et al*.)^35^. X-ray fluorescence spectra was used and explained the lamellae formation in the clay fractions of golf course samples (Glen R. Obear, *et al*.)^36^. Additionally, some commercial activities and industry activities also have influence on the golf course. Extremely high values of the pollutants such as Pb, Zn, Cu and As using PXRF approach were found during the investigation of urban soils in Galway City, Ireland^37^. Golf course located in Amarillo, some commercial activities and traffic have influences on soil heavy metal accumulation, and resulted into the uneven spatial distribution of Sr concentration in the golf course. These high concentration areas of PXRF-elements indicated the dominant role of anthropogenic activities as the major sources of heavy metals in soils (MengYang, *et al*.)^38^. The case study also showed that anthropogenic activities on golf course profoundly affected Sr concentration and variation, turfgrass in the golf courses were frequent with anthropogenic activities, which increased the spatial variability of PXRF-quantified Sr element^39^.

There were very high Fe and Mn concentrations available around pH values of 3.5 located in mining soils, but the underlying spatial couple relationship between pH, Fe and Mn wasn’t revealed^40^. In particular, the contributing factors on the PXRF spectrum of Fe concentration were explored to increase the accuracy of field PXRF measurements^41^. Only limited on certain linear relationship, there was few spatial distribution models relevant to the PXRF-quantified elements and soil properties. In monitoring metal pollution in soils using the PXRF method, satisfactory correlations were obtained between the AR (Aqua Regia) and the PXRF-quantified concentrations of Ca, Cu, Cr, Ni, Pb and Zn^42^. Strong linear correlations were found between As, Ca, Cr, Cu, Fe, K, Mg, Mn, Ni, P, Pb, Si, Ti and Zn^43^. Although the above studies explored more or less the correlations between heavy metals using PXRF quantification, or other methods, the spatial regression model was not established between soil properties and PXRF-quantified elements. While in this study, a new OLSM model was constructed between pH and EC, silt, PXRF quantified elements (K, Ca and Sr), which provided the beneficial enlightenment for the contributing factors to clarify the influence of soil properties on the PXRF spectrum. OLSM was used to evaluate relationships between two or more feature attributes or variables, a set of diagnostics that examines some checks improves the prediction accuracy of the model. The OLSM calculates a coefficient and performs a statistical test to determine whether that variable is helping model or not. Generally, some checks were verified and done for model performance. Such as, some of regression explanatory variables are statistically significance, model basis affects the predicted results, the adjusted R^2^ value and R^2^ value are also an important measure. If the OLSM has gone through the above checks and met all the necessary criteria of verification parameters, we think that how well the model explains the relationship between explanatory and dependent variables. Actually, in the study, we analyzed all of the variables from lab measured variables and PXRF-quantified elements, only pH = −0.00063EC − 0.01169silt − 0.00022K − 0.00003Ca + 0.00316 Sr + 8.4576 has gone through the checks of model parameters and statistical significance, met the all the necessary criteria of parameters. Therefore, the quantitative relationship is revealed between lab measured variables (pH, EC and silt) and PXRF-quantified elements (K, Ca and Sr).

Geostatistics, which is a well-estimated scientific discipline that provides flexible spatial analysis methods to quantify uncertainties about the contaminant concentrations of PXRF-elements in this context (D’Or *et al*.)^44^, but geostatistics practice easily triggers smoothing effects (Yujian, Yang, *et al*.)^45^, raises overestimated value and underestimated value of PXRF-quantified elements. While the EBK avoids smoothing effects, it provided a more practical spatial statistical tool with being automated, intelligence and better accuracy. Detailed maps from PXRF quantification integrated with EBK should be useful in detecting parts of fields with particularly high or low risk of PXRF-quantified elements in golf course soils. Previous studies also showed that more quantitative delineation of soil mapping prediction accuracy and uncertainty was the key challenge remaining according to qualitative criteria and renders maps^46^. Additionally, agricultural and recreational practices greatly altered the distribution balances of soil properties, responses and feedbacks^47^. Evidently, spatial analysis has become an important tool of quantitative delineation of soil properties, while PXRF quantitatively better responded to heavy metals concentrations in soil, spatial mapping of soil properties and PXRF quantified-elements by EBK is a better way to help precisely and well understand the spatial variability over the golf courses, and optimize turfgrass decision support and improve golf course management. Therefore, spatial analysis integrated with PXRF will play an increasingly key role of soil mapping uncertainty, responses and feedbacks, which also offers a unique opportunity to address soil pollution and remediation^48^.

In summary, sampling, scanning, predicting, mapping and spatial regression model were completed and extended the application of PXRF spectrometry combined with spatial analysis to the rapid probing soil elements. The integration of PCA, EBK and OLSM also provided better perception of the series of studies for the pedogenesis causes, spatial variability and spatial relationships of the PXRF-quantified elements and soil properties in golf courses soils. The preliminary classification of PXRF-quantified elements was formed by PCA, the spatial variability characteristics of PXRF-quantified elements and soil properties was in detail delineated by statistics combined with EBK, the loosely couple model between lab variable (pH, soil textures) and PXRF-quantified elements (Sr, K, Ca) was constructed in the study. However, study results are still lack of sufficient proofs supporting the tightly couple model between soil properties and PXRF-quantified elements in golf courses soils. Therefore, future work is to develop the further study via the investigation of golf course soil and turfgrass with different soil textures, different climate, and different irrigation management practices, etc. In addition, one possible future extension of whether the principal component of Zn, Ti, Fe, Rb, V, Mn and Zr could be originated from the similar lithology origin, or the principal component of Sr element concentration was more likely linked to the contaminants from anthropogenic activities (e.g., traffic, management practices), needs further verification by additional evidence, or measured data.

## Materials and Methods

### Experimental sites and soil sampling

**Figure 8:**
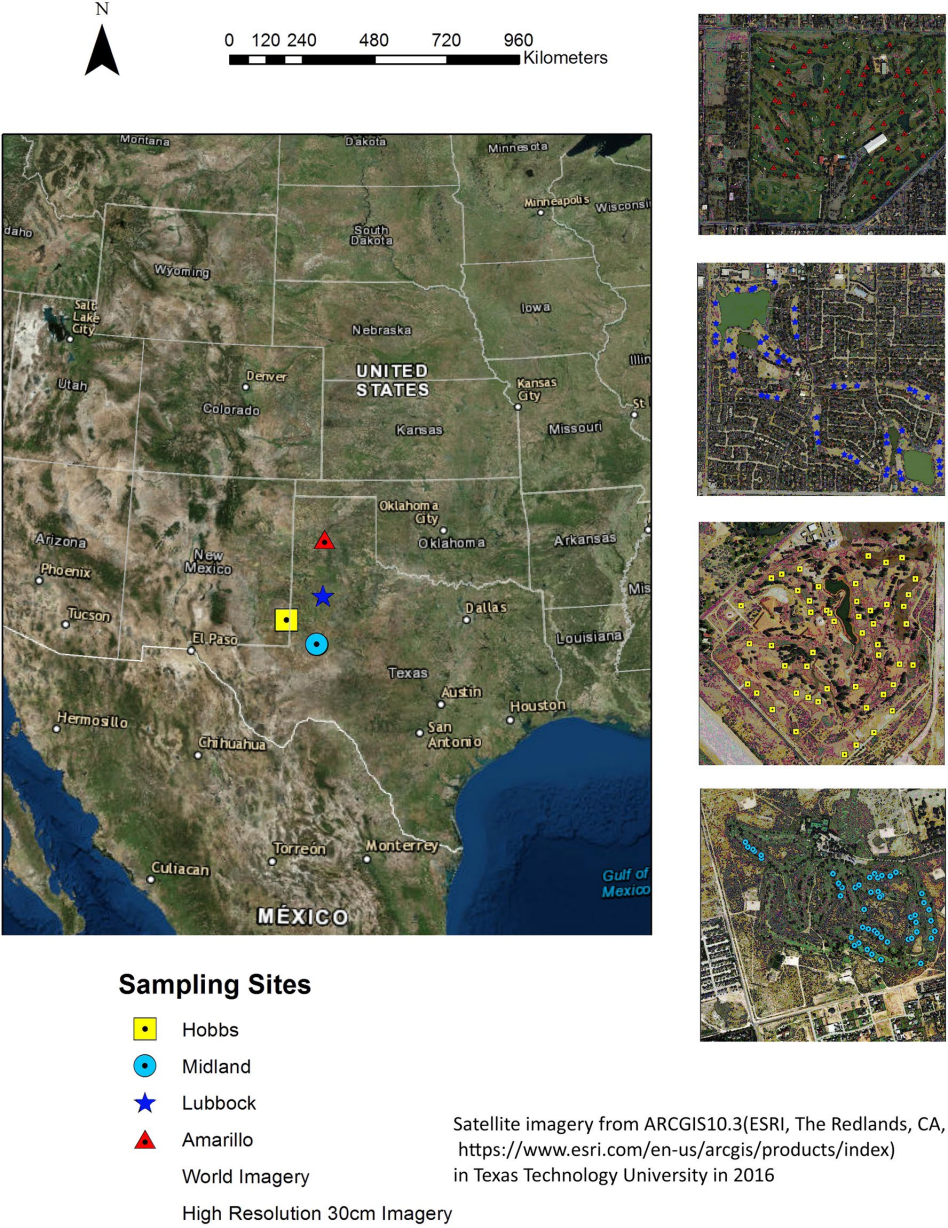
Sample sketch map of 4 golf courses in Amarillo, Hobbs, Lubbock and Midland, U.S.A.

The soil sampling study was conducted in 4 golf courses. Three of them are in the Southern High Plains of western Texas, respectively at Lubbock, Amarillo and Midland, and another one at Hobbs in eastern New Mexico. Totally 200 samples were collected in 4 golf courses, 50 of which were collected in each golf course, in 2016. The sampling points are mapped in Fig. 8 using ArcGIS 10.3 platform (ArcGIS 10.3 (ESRI, The Redlands, CA)) provided by Texas Tech University. The total area of 4 golf courses is approximately 130000 km^2^ and characterized by semi-arid climatic conditions, having hot summers and mild winters with occasional strong cold fronts and a gentle west-to-east gradient of increasing precipitation. Mean annual precipitation ranges from about 300 to 500 mm along this gradient, and there is significant inter-annual variability. Amarillo lies on the north-eastern edge of the southern high plains, while Midland lies on the southeastern edge of it. Hobbs lies on the west of Midland, and Lubbock lies in the middle of the high plains. For example, the golf course contains 10 to 12 ha of irrigated fairways in the Lubbock golf course, which were planted with hybrid bermudagrass (Cynodon dactylon (L.) Pers. × C. transvaalensis Burtt-Davy)^49^.

### Field and laboratory methods

A total of 200 surface soil samples (0–15 cm) were collected using a simple handheld trowel^50^. Vegetation was gently scraped aside and ~150 g of soil was placed in a sealed plastic bag for transport to the laboratory. Each collection location was geo-located with an E-trex(Garmin, Olathe, KS) global positioning system (GPS) receiver. Importantly, randomized or equidistant sampling schemes were not possible given the layout of the golf course. Soil samples were obtained from fairways only, resulting into somewhat linear sampling across the property. The courses followed a tortuous path between homes, streets, and businesses, which were identical to the practical situations. Soil analyses were conducted in the Texas Tech University Pedology Laboratory in Lubbock, Texas. In the lab, samples were air dried and lightly grounded to pass to a 2 mm sieve prior to all other analyses. Soil electrical conductivity (EC) was measured in a 1:2 solid (soil) to water suspension^51^ using a traceable digital salinity meter. Soil pH was determined on saturated paste per Soil Survey Staff^52^. Pastes were allowed to equilibrate for 24 hours, and then quantified using an Orion 2 Star pH meter (Thermo Scientific, Waltham, MA). Mehlich III extractable elements were obtained^53^. Particle size analysis was accomplished using a model 152-h hydrometer^54^. Clay determination was made at 1440 min and sands were sieved using a 53 µm sieve.

### PXRF analysis

All collected soil samples were scanned with a DP-6000 model PXRF (Olympus, Waltham, MA) with deference to PXRF-quantified K, Ca, Zn, Ti, Fe, Rb, V, Mn, Zr and Sr in each sample. The instrument was equipped with a Rh-X-ray tube energized at 10–40 kV with integrated silicon drift detector (165 eV) for optimized measurement of light elements. The instrument was operated in Soil Mode whereby each of three beams scan the soil sample sequentially for 30 sec each. Thus, the total scan time was 90 sec per sample. The beams ensure full coverage of elemental detection, with each beam or combination of beams are detected different groups of elements. Calibration of the instrument was conducted using a 316 alloy clip tightly fitted over the aperture. Each soil sample was scanned in triplicate, with the PXRF unit physically repositioned between scans such that an average value was reported^55^. The PXRF instrument was operated in “Soil Mode” capable of detecting the following suite of elements: Sr, Zr, Mo, Ag, Cd, Sn, Sb, Ti, Ba, Cr, Mn, Fe, Co, Ni, Cu, Zn, Hg, As, Se, Pb, Rb, P, S, Cl, K, Ca, and V.

### Statistical and geostatistical methods

All statistical analyses including descriptive procedure, Pearson’s correlation matrix and principal component analysis (PCA) were conducted using SPSS Statistics v23 (IBM, Armonk, NY). In general, dimensionality reduction loses information, but PCA-based dimensionality reduction tends to minimize that information loss, which results from the following concrete calculation steps, inputting the covariance matrix of variables, calculating the eigenvectors and eigenvalues of the covariance matrix, sorting the corresponding eigenvectors in descending order, and deriving the new predictors. PCA is used to obtain the initial factor solution, PCA-based calculation presents the first component which has maximum variance, and successive components explain progressively smaller portions of the variance and are all uncorrelated with each other. We performed two separate principal components analyses (PCA), thus, PXRF-quantified elements including Zn, Ti, Fe, Rb, V, Mn, Zr and Sr were grouped into a two-component model by PCA dimensionality reduction in golf courses soils^56^. The descriptive procedure displays univariate summary statistics for some variables in a single table. The calculation of soil variables was performed to include arithmetic means, standard deviation, coefficient of variation, minimum and maximum, and skewness and kurtosis. Pearson’s correlation matrix is a 2D array with numbers that describes the degree of relationship between any two variables. The matrix is one of the most commonly used statistics to describe the degree of relationship between soil properties and PXRF-quantified elements.

OLSM was used to evaluate the spatial relationship between soil properties and PXRF-quantified elements. OLSM not only provided an optimal model between the variables you were trying to understand, but also created a single regression equation to represent the process the variables drive. More importantly, OLSM generates predictions according to a dependent variable in terms of its relationship to a set of explanatory variables. In order to further understand and quantify the coupled relationship between typical soil properties and PXRF-quantified elements, we performed OLSM linear regression to model a dependent variable in terms of its relationships to a set of explanatory variables.

Spatial variability of soil properties and PXRF-quantified elements was analyzed and OLSM modeling was performed using ArcGIS 10.3 (ESRI, The Redlands, CA). EBK was employed as an appropriate technique for modeling the spatial distribution of soil properties and PXRF-quantified elements. Generally, some important parameters were used to evaluate prediction accuracy and uncertainty, including mean error (ME), mean standard error (MSE), average standard error (ASE), root mean square error (RMSE), mean standard error (MSE),and root mean square standardized error (RMSSE). ME of the model is equal to 0, ASE is equal to RMSE, MSE is equal to 0, and RMSSE is equal to 1^57^, which indicates the perfect “goodness of fit” accuracy of the EBK approach.

## Data Availability

All data included in this study are available upon request by contact with the corresponding author.
